# Forecasting Zoonotic Infectious Disease Response to Climate Change: Mosquito Vectors and a Changing Environment

**DOI:** 10.3390/vetsci6020040

**Published:** 2019-05-06

**Authors:** Andrew W. Bartlow, Carrie Manore, Chonggang Xu, Kimberly A. Kaufeld, Sara Del Valle, Amanda Ziemann, Geoffrey Fairchild, Jeanne M. Fair

**Affiliations:** 1 Los Alamos National Laboratory, Biosecurity and Public Health, Los Alamos, NM 87545, USA; abartlow@lanl.gov; 2 Los Alamos National Laboratory, Information Systems and Modeling, Los Alamos, NM 87545, USA; cmanore@lanl.gov (C.M.); gfairchild@lanl.gov (G.F.); sdelvall@lanl.gov (S.D.V.); 3 Los Alamos National Laboratory, Earth Systems Observations, Los Alamos, NM 87545, USA; cxu@lanl.gov; 4 Los Alamos National Laboratory, Statistical Sciences, Los Alamos, NM 87545, USA; kkaufeld@lanl.gov; 5 Los Alamos National Laboratory, Space Data Science and Systems, Los Alamos, NM 87545, USA; ziemann@lanl.gov

**Keywords:** infectious disease, zoonotic, mosquito, vector-borne, climate change, range expansion, epidemiology

## Abstract

Infectious diseases are changing due to the environment and altered interactions among hosts, reservoirs, vectors, and pathogens. This is particularly true for zoonotic diseases that infect humans, agricultural animals, and wildlife. Within the subset of zoonoses, vector-borne pathogens are changing more rapidly with climate change, and have a complex epidemiology, which may allow them to take advantage of a changing environment. Most mosquito-borne infectious diseases are transmitted by mosquitoes in three genera: *Aedes*, *Anopheles*, and *Culex*, and the expansion of these genera is well documented. There is an urgent need to study vector-borne diseases in response to climate change and to produce a generalizable approach capable of generating risk maps and forecasting outbreaks. Here, we provide a strategy for coupling climate and epidemiological models for zoonotic infectious diseases. We discuss the complexity and challenges of data and model fusion, baseline requirements for data, and animal and human population movement. Disease forecasting needs significant investment to build the infrastructure necessary to collect data about the environment, vectors, and hosts at all spatial and temporal resolutions. These investments can contribute to building a modeling community around the globe to support public health officials so as to reduce disease burden through forecasts with quantified uncertainty.

## 1. Introduction

The epidemiology of infectious diseases is constantly fluctuating in response to environmental changes and changing interactions among hosts, reservoirs, vectors, and pathogens. This is particularly true for zoonotic diseases that infect humans, animals of veterinary importance, and wildlife. Within the subset of zoonotic diseases, vector-borne pathogens are changing more rapidly with climate change and potentially have a more complex epidemiology. In the past 80 years, the majority of global emerging infectious diseases have been zoonotic [1]. While most of the zoonoses arise from wildlife (72%), vector-borne emerging diseases are increasing at a more rapid rate [1]. Along with an increase in emerging zoonotic diseases, there have been range expansions of reservoir hosts, vectors, and the pathogens they harbor.

Vector-borne diseases account for more than 17% of all infectious diseases, causing more than 700,000 deaths annually [2,3]. For example, almost 4 billion people in over 128 countries are at risk of contracting dengue, with 96 million cases estimated per year [2,4]. Dengue virus has both animal hosts and reservoirs that are both impacted and help maintain virus circulating in populations [5]. Many of the vectors that transmit important zoonotic infectious diseases are bloodsucking insects that ingest disease-producing microorganisms during a blood meal from an infected host and then later inject it into a new host during their subsequent blood meal. Mosquitoes are the best-known disease vector. However, other vectors, such as ticks, black flies, sandflies, midges, fleas, and triatomine bugs are also important vectors of human pathogens. Table 1 lists the primary zoonotic arboviruses and other pathogens vectored by Dipterans, and Table 2 lists the primary animal (non-zoonotic) pathogens vectored by Dipterans. For a recent review on tick-borne pathogens, see [6]. The rest of this review focuses on modeling mosquito vectors and mosquito-borne infectious diseases in response to climate change.

Mosquitoes are heavily dependent and closely tied to the environment [7,8]. A mosquito’s life is a microcosm of water, available habitats, temperature, predators, and competitors. Each aspect of a mosquito’s life history is greatly influenced by even the slightest changes in the environment in unpredictable ways. For example, droughts can increase mosquito habitat by increasing stagnant water in streams, thereby increasing mosquito populations [9]. In other cases, range expansion is simply due to warmer winters in the northern latitudes [10,11].

## 2. Changing Mosquito Vector Biology and Range Expansion

Emerging infectious diseases are directly influenced by changing environmental conditions [1,51]. The results of these geographic expansions and range shifts represent major health crises in many parts of the world [52,53]. Vector-borne infectious diseases make up a significant portion of zoonotic diseases, which have increased in the last few decades [1]. Mosquitoes shift and expand their ranges into new areas as environments change and become more suitable, bringing the pathogens they harbor with them. Mosquitoes—important vectors for many infectious diseases of human importance—are sensitive to environmental conditions, especially temperature and precipitation [7]. As aquatic insects, their life cycle and developmental time depends on water availability [54]. Temperature is also important for their development time and their ability to overwinter [55]. Being able to overwinter due to warmer winter temperatures may aid in the northward expansion of many species [10,11]. In addition, increased temperatures induce faster development times [7,56], which may be especially important in arctic environments [57].

Most mosquito-borne infectious diseases are transmitted by mosquitoes in three genera: *Aedes, Anopheles, and Culex*. Species in the genus *Aedes*, particularly *Ae. Albopictus* (Asian tiger mosquito) and *Ae. aegypti* are vectors for a number of zoonotic diseases, including dengue virus, chikungunya virus, Zika virus, and yellow fever. *Anopheles* mosquitoes are responsible for transmitting malaria and a few other pathogens, such as canine heartworm (Dirofilaria immitis) and species that cause filariasis (e.g., Wuchereria bancrofti). *Anopheles gambiae* and *An. arabiensis* are the most responsible vectors for malaria transmission in Africa. *Culex* mosquitoes, predominantly *C. pipiens, C. tarsalis, and C. quinquefasciatus*, carry and transmit West Nile virus and Saint Louis encephalitis virus. Mosquitoes in all three genera have experienced range expansions [58,59,60,61,62,63,64,65]. *Aedes aegypti* is the most widespread, with an almost global distribution through repeated invasions [66]. As mosquito species expand their geographic distributions, the diseases they harbor are expanding and are predicted to continue to expand [67], representing new challenges for places previously not affected. For example, chikungunya virus has spread from Africa and Southeast Asia to the subtropics and the western hemisphere [68], Zika virus has spread rapidly from Africa to the Americas [69], and West Nile virus has spread to British Columbia, Canada [70].

Two species of mosquitoes in the genus *Aedes* have experienced the most range expansion globally—*Aedes albopictus* and *Ae. aegypti* [66]. *Aedes albopictus* is the most invasive mosquito species in the world [71], spreading from southeast Asia to every continent except Antarctica [72]. Global air and sea travel [73] and the used tire trade [72,74,75] have been proposed to be major causes of the worldwide dispersal of *Ae. albopictus*. Eggs of this species are long-lived, desiccation-resistant [76], and respond to shorter, colder days by going into diapause [72,74]. *Aedes aegypti* spread from Africa to the tropics and subtropics around the world by way of human movement [77,78]. In addition to climate impacts on vector distributions, urbanization may exacerbate the invasive potential of vectors following the colonization of new areas [66]. Both *Ae. albopictus* and *Ae. agypti* have urban and suburban habitat preferences, and the habitats of larval development include man-made containers [76].

Species of *Anopheles* and *Culex* mosquitoes have also increased their geographic range [58,59,70], but have not become as invasive as the two species of *Aedes*. The increase in *Anopheles* mosquitoes, mainly *An. arabiensis*, may be due to their ability to resist desiccation and survive severe dry seasons, resulting in perennial transmission of malaria to humans [79,80,81]. However, *Anopheles gambiae* has also been shown to transmit malaria continuously during the dry season [80] and expand their niches into marginal habitats [61], both of which may act to increase their geographic range in the future [82]. One of the most important malaria vectors in the neotropics (Central and South America) is *An. darlingi*, and is predicted to undergo range expansions [62,83].

The *Culex pipiens* complex, made up of *C. pipiens sensu stricto* and *C. quinquefasciatus*, is distributed worldwide. *Culex quinquefasciatus* is most prevalent in the tropics and subtropics, while *C. pipiens* is found in temperate climates [84]. Recently, a new species of concern, *C. coronator*, was discovered in the southwestern United States [85,86], and as far north as Virginia [87]. This species has expanded its range from the America subtropics and tropics [85]. Importantly, this species has been documented harboring several arboviruses of human importance [86]. Other *Culex* species are experiencing range shifts northward into Canada [64,70]. Both *Culex pipiens* and *C. quinquefasciatus* are predicted to spread further northward in Canada [64,88].

Mosquito responses to environmental conditions are variable and often stray from predictions from models that take into account climate to determine future distributions. For example, when *Ae. albopictus* invaded new continents (e.g., South America), it resulted in niche shifts, which may be due to adaptive genetic changes or result from founder effects [63]. The ability to shift niches upon invading new areas may be one reason why *Ae. albopictus* is so widespread. Additionally, several species have been shown to become more tolerant to saline and brackish waters. These species include *Anopheles sundaicus*, *An. culicifacies*, *An. stephensi*, *Aedes aegypti*, *Ae. Albopictus*, and *Culex sitiens* [89]. Salinity tolerance may be important to consider in studies predicting future mosquito distributions because brackish and saline water along coastlines is predicted to increase [90]. Hybridization is another ecological factor that may determine the extent of species distribution and influence range expansion of certain species (reviewed in [91]). For example, *An. gambiae* gained a critical gene from *An. arabiensis* allowing it to move from rainforests to drier habitats, such as savannahs [92,93]. Further research is needed to understand how hybridization may contribute to range expansion in other mosquito genera. Predicting shifts in range distributions of mosquito species is challenging and requires a comprehensive modeling approach that incorporates vegetation modeling, hydrology, epidemiological modeling, human movement behavior, mosquito behavior, and host, vector, and pathogen biology and evolution.

Mosquitoes are closely tied to and track environmental conditions because their life cycle depends on environmental conditions. Mosquito expansion will likely continue well into the future because of increased temperatures, greater probabilities of overwintering, and changes to precipitation regimes. Although climate change directly influences mosquito distributions through changing environmental conditions, climate change can also impact distributions indirectly. Humans respond to climate change by altering their surroundings. For example, drought has caused southeast Australian communities to install water storage tanks, which is predicted to increase the distribution of *Ae. aegypti* and increase the risk of emerging and re-emerging diseases in these areas [94]. Changes in human movement will also affect mosquito dispersal and impact future distributions. Predicting range expansions of mosquito vectors and mosquito-borne diseases therefore needs effective climate, human, and epidemiological modeling, which when combined, will provide more accurate forecasts for vector and disease risk.

## 3. Epidemiological Modeling of Vector-Borne Infectious Diseases

While yearly dengue epidemics continue [4,95,96], the chikungunya virus and Zika virus have recently emerged and caused large outbreaks in the Americas, spreading rapidly across the continent after introduction and causing long-term effects, including Zika-related birth defects. Chikungunya virus infected upwards of 30% of vulnerable populations in South and Central America. While the full effects of Zika virus are still being understood, recent sero-surveys in Brazil indicate a more than 60% attack rate [97,98,99,100]. It is not clear if chikungunya virus and Zika virus will continue to expand geographically and become endemic. In addition, climate change and globalization have increased the potential for wider spread of vector-borne diseases [101]. Thus, there is an urgent need to study these diseases in different regions and to produce a generalizable approach capable of mapping risk and forecasting outbreaks to alert vulnerable populations and inform decision support while increasing scientific understanding [102].

Prior research has primarily focused on understanding outbreaks at limited spatial and temporal scales [103,104,105,106,107,108,109,110]. This is because outbreak size, timing, and relationship to exogenous factors varies widely across time and space [105,106,111,112,113,114,115,116] and because reliable data directly related to outbreak risk (e.g., case counts, biting rates, mosquito infection rates) is hard to come by. Data coverage as well as team expertise is also incomplete. Previous studies have focused on analyzing a single or small number of satellite images over one region [104,106,110,117,118,119,120] or analyzing a subset of data streams such as weather or Internet data to study vector-borne disease dynamics [121,122,123,124,125,126,127,128]. These provide a limited picture and sometimes-conflicting results [105,110,129,130,131,132] (e.g., role of El Niño). Modeling of Infectious Disease Agents (MIDAS) groups have focused on smaller spatial-scale transmission (<200 m transmission chains [133]) for Thailand and on mechanistic stochastic models with dependence on temperature and other factors at continental or global scales [134]. Other teams have used ecological niche modeling to produce static maps of suitability (not risk per se) for Zika, dengue, and chikungunya and their associated vector species, *Ae. aegypti* and *Ae. albopictus* [95,135,136,137,138].

Ecological studies have demonstrated that certain variables such as Normalized Difference Vegetation Index (NDVI), precipitation, and temperature can predict the severity of mosquito-borne disease transmission months in advance [110,129,139]. These variables have been used to predict disease risk such as West Nile virus in the United States [140]. Mosquito-borne disease modeling has been limited to either local studies that consider selected fine-scale key processes or to larger-scale models that have a limited representation of key processes and their interactions. For example, some process-based models consider the impact of temperature [141,142,143,144,145,146,147,148] or rainfall [147,149,150,151] on mosquito lifespan and development rates without explicitly considering the nonlinear response of mosquito habitat to weather (i.e., formation and persistence of standing water in the landscape). Recent modeling studies (e.g., malaria [24,152,153] and Rift Valley fever [154]) have begun to consider the linkage to hydrology and climate. But they are either limited to small scales (e.g., watersheds or small regions) [153,154] or are based on a simple calculation of water balance without considering real land surface characteristics [152]. Meanwhile, statistical models built on the relationship between local meteorological/environmental factors, mosquito physiology, and/or reported disease cases have been developed for understanding the ecological niches of mosquito and mosquito-borne diseases [78,95,116,155,156,157,158]. Because they are trained solely on past observations, they generally do not capture the coupled nonlinear processes (e.g., vector range expansion and human-mosquito contact) [112,148] in conditions not yet encountered (i.e., “no-analog” and beyond the space of available data for model calibration) resulting from weather extremes, climate, and socioeconomic changes.

Laboratory and field studies have shown relationships between weather/climate and risk for mosquito-borne diseases (e.g., Rift Valley Fever) [159], tick-borne diseases [160], and other vector-borne diseases. Several research teams, including ours, have incorporated these factors into working models [161,162,163]. A recent systematic review by the National Exposure Research Laboratory [160] highlights the need for a “rigorous multi-system modeling approach to improve our knowledge about the important mosquito vector, *Aedes* spp. presence/abundance response to the interaction between environmental, socioeconomic, and meteorological systems”. The current state of the art has focused on particular pathogens in particular regions using a subset of the needed factors [160,164,165,166]. The few global or continental models that exist focus on a single pathogen [137,141,163] or on ecological niche models rather than explicit disease dynamics [137].

## 4. Earth System Modeling: The State of Climate Forecasting

The modeling of global climate systems dates back to 1960, and were based on weather prediction models (e.g., [167]). Most of these early models only include atmosphere without detailed representation of land (e.g., no explicit representation of vegetation) and ocean process (e.g., motionless ocean). However, there are also pioneering models that coupled atmosphere and ocean currents [168,169]. Because these models capture the circulation of both atmospheric and ocean currents, they are referred to as general circulation models (GCMs). With the advancement in computing powers and our improved understanding of coupled physical systems, GCMs have made great progress in simulating the coupled physical climate processes (e.g., winds, clouds, land surface, oceans, and ice) and atmospheric chemistry, aerosol, and static vegetation in our Earth system from the 1960s to 2000s [170]. Since the late 2000s, Earth system models (ESMs) began to evolve from GCMs to simulate the interaction between climate and biogeochemical components (e.g., dynamic terrestrial vegetation and ocean biogeochemistry) to better simulate the feedback of biological systems to our climate [171]. Meanwhile, great progress has been made to integrate the human component into ESMs (i.e., the integrated ESM or IESM) [172]. The modern ESMs and IESMs provide the potential to simulate zoonotic relevant components including biological components (e.g., vegetation dynamics), abiotic components (e.g., inundation and water temperature), and human components (e.g., urban environment and population changes). All these progresses provide great potential for seamless coupling with disease modeling for a better prediction of disease risks under population growth, warming, and climate extremes.

## 5. Bridging the Gap Between Epidemiological and Earth System Modeling

Although great strides have been made in simulating and predicting global climate with large-scale ESMs, coupling these forecasts with human and living-natural (HLN) systems is critical for planning and mitigating long-term impacts of changes in the environment. HLN systems interact nonlinearly with climates, and data that account for relevant parameters and states of system dynamics in a single source are limited, posing a significant challenge in understanding and predicting the response of these systems. Mosquito-borne diseases are considered a HLN system with rich traditional (e.g., number of cases) and non-traditional (e.g., remote sensing imagery) data streams. Mosquito abundance and virus development rates show both inter- and intra-seasonal variation, and are affected by long-term changes in climate regimes leading to potential range expansion of both mosquitoes and associated diseases. Changes in temperature, climate variability, and extreme weather events are already impacting mosquito-borne diseases around the world [173,174,175]. A recent example is the emergence of Zika virus throughout Latin America, which was likely fueled by the hot drought during the 2015–2016 El Niño [175]. The mosquito-borne disease HLN system not only affects human health in the US and troops abroad, but is also linked to poverty and regional stability.

**Figure 1:**
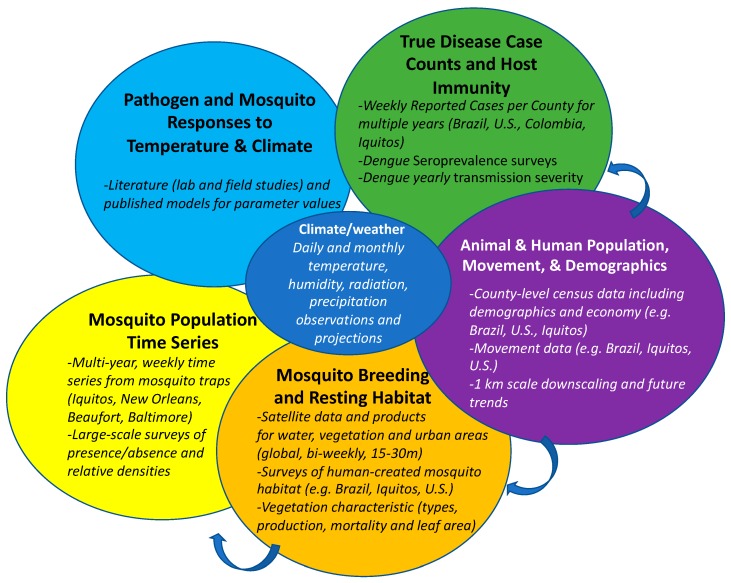
Data requirements to bring together climate and epidemiological models and ground truth real-time data. Arrows indicate data that can be used to inform additional data categories. Bold represents Data Categories and italics are representative subsets of Best Available Data.

We face three key challenges to be able to make reliable large-scale predictions of disease risks at both short-term (seasonal/sub-seasonal) and long-term (decadal) temporal scales, which is critical for planning and improving disease mitigation strategies. First, there is currently no capability to integrate key processes at large scales including (1) hydrology and vegetation that affect mosquito habitats; (2) temperature and humidity that affect mosquito population dynamics and pathogen replication; and (3) human population density and movement that affect vector human contact and facilitates the dispersal of mosquitoes and the development of additional mosquito habitats. There are numerous baseline data requirements for both the epidemiological and climate models for zoonotic infectious diseases (Figure 1). Statistical models built on the relationship between local meteorological and environmental factors and reported disease cases have been developed for understanding the ecological niches of mosquito-borne diseases [116,155,156]. However, they do not directly incorporate the coupled nonlinear processes (e.g., human behavior or mosquito population dynamics) [112], a weakness under novel future climate conditions. Meanwhile, most current process-based models have only incorporated the impact of temperature [141,142,143,144,145] and rainfall [149,150,151] on mosquito population dynamics without explicitly considering the formation and persistence of water on the landscape, which is critical for accurately predicting the nonlinear response of mosquito habitats to precipitation. One exception is the HYDREMATS model [153], but it is limited to local watersheds. Thus, it cannot account for key processes of disease transmission such as human movement or nearby pathogen reservoirs.

**Figure 2:**
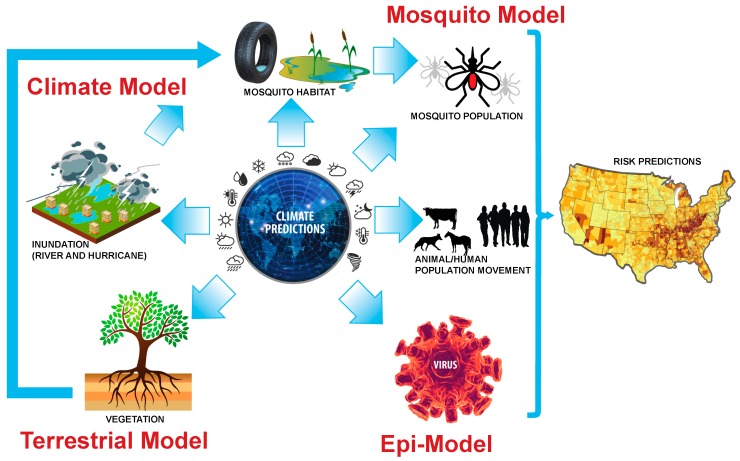
Disease and climate systems for mosquito borne diseases. Each system must be coupled together with validation from ground truth real-time data. Data from Figure 1 feeds into each of these systems and data fusion issues are addressed throughout the process.

Second, although recent models have started to incorporate human population and movement data [141,176,177], they have not yet considered future human populations and movement that are relevant to predicting disease risk in the future. While animal movement data is also required, it can be either easy or impossible to obtain, based on the region. Third, although field and lab studies and analysis of diverse and often non-traditional data streams critical to developing and validating coupled HLN-climate systems are increasingly available, the research community lacks an advanced data fusion and mining system to extract critical information from different data sources for parameterization, development, and evaluation of such large-scale, complex models. The climate system, resulting environmental change, vectors, animal and human hosts, and infectious pathogens are each in its own a complex system. Data requirements, existing models, a foundational understanding of infectious diseases, statistical and data fusion, and computer science are all required for predicting how climate change may impact vector-borne diseases (Figure 2). The main hurdle to the above three challenges can be attributed to the lack of large investments that enable seamless collaboration between diverse and highly skilled subject-matter experts, availability of computational resources to scale up and integrate key processes within the complex systems, and data fusion/integration approaches needed to validate models while capturing uncertainty in model parameters, inputs, and predictions.

## 6. Data Fusion and Data Requirements

Data fusion is necessary to understand the complex processes of vector-borne diseases. Fusing heterogeneous data provides a way to link the data model, the process model for the disease dynamics (including transmission rates), and R_0_, as well as account for varying levels of spatial resolution [178]. Each of these components has been well-established but have not been integrated together in a disease model. Additionally, it can be a challenge to fuse data with a mechanistic understanding [179,180,181]. Due to the complexity of modeling in both the process and the data, researchers generally focus on either a model-based (mathematical) or data-based (statistical) approach to understand predictions of ecological dynamics [182]. However, both the data and the mechanistic process underlying the physical process are often of interest in disease modeling. Therefore, it is not enough to fit a statistical model to observations or only use a model-based approach that ignores the data [123]. There are several statistical challenges associated with disease modeling, including modeling and estimating coupled systems, dealing with unobserved variables, and modeling the spatial-temporal dynamics [183]. Statistical inference for time-series models with incomplete data and for process models can be particularly challenging as they may be computationally expensive and oftentimes intractable. Stochastic models, on the other hand, add flexibility to the model to fit real data. This provides a framework where parameters can be identified even when they can be unidentifiable in a deterministic setting. It focuses on the distribution associated with the characteristic of a process. However, it does not incorporate all sources of data from climate, water sources, and transmission.

A particular challenge present in vector-borne disease modeling is that the underlying phenomena are generally difficult or impossible to directly measure. In an ideal situation, knowledge of mosquito population distributions through time, broken up by species and then coupled with human population distributions through time, would be used to derive risk maps. This knowledge could directly inform a mechanistic or statistical model to provide high-fidelity forecasts. However, mosquito population distributions are difficult to capture. Ball et al. [184] are developing an autonomous sensor that uses passive sugar baiting to lure mosquitoes in order to test their saliva for the presence of mosquito-borne viral pathogens [184], but these sensors are still in their infancy, and deployment at the scale of a large city, state, or country is not yet possible. As a result, no large-scale mosquito population distribution data through time exist. This gap, along with complications coming from the structure of modern disease surveillance systems, has resulted in a revolution in the disease modeling literature in which *proxy datasets* are used to fill in the disparity in our knowledge. Such datasets indirectly relate to the phenomenon at hand. For example, internet data (e.g., [185,186,187,188]) have been used to complete the gaps in our public health data; searches for specific disease-related keywords such as cough or fever, for instance, tend to highly correlate with actual disease incidence. Using knowledge of the mosquito lifecycle (i.e., that mosquitoes require certain temperature and precipitation conditions for breeding), proxy datasets such as weather, demographics, satellite imagery, and other related datasets can be used to provide additional insight into mosquito locations. The underlying hypothesis to these data fusion approaches is that, while no individual proxy dataset will provide enough information to accurately forecast disease incidence, the fusion of *multiple weak indicators* derived from disparate data streams will provide a more complete picture.

Once this approach is considered, however, the complexity of the data fusion problem greatly increases; it may not be intuitive, for example, how internet data coming from Wikipedia and spectral signals coming from satellite imagery could be fused. Furthermore, they will relate to disease incidence on different timescales, e.g., healthy vegetation in satellite imagery (as a proxy for standing water) will be a multi-week leading indicator [189], whereas Wikipedia access logs will be closer to a real-time indicator. Another factor is that each data source has the potential to have large measurement errors, but because the data types include hard (e.g., physics-based phenomenologies) and soft (e.g., text) data sources, the mechanics of these errors are highly variable. Because each of the disparate data streams is important for forecasting and understanding disease dynamics, it is equally as important to appropriately account for leading/lagging indicators, error propagation, etc., when building the fusion models. The conditional model, for instance, is flexible as it accounts for the uncertainty in the data based upon prior knowledge of the data parameters and characterizes the relationships in the data and the inherent latent structure. The process model spatial and temporal rate variations are incorporated directly as an additional layer in the hierarchical model.

An added complexity of data fusion is that the sources for climate and mosquito data are generally inherently on different spatial and/or temporal scales, e.g., fine-scale data as in mosquito habitats, and coarse gridded data as in climate data. Oftentimes, the sources of data are spatially misaligned, meaning the data are on different grids. To account for the misalignments, a spatial interpolation method, such as kriging, can be used [190]. Additionally, if the data layers are nested or data layers overlap (non-nested), spatial modeling approaches exist to account for the misalignment in the spatial grids [191]. A hierarchical or multilevel model structure accommodates different spatial aggregations, as well as allowing for shared characteristics among the groups [192,193]. A conditional modeling framework, i.e., a Bayesian or maximum likelihood approach, can combine multiple processes and datasets in an analysis [194]. The conditional model provides a way to pool data from multiple sources and weight them based upon the content of the information.

## 7. Assumptions or Challenges of Disease Forecasting and Modeling in Animals and Humans

There are three major challenges in achieving the scientific and technological advances made in weather forecasting and transferring them into disease forecasting; these are: (1) data availability, (2) human behavior, and (3) funding [123]. In terms of data availability, unlike weather forecasting, there is the lack of real-time ground truth data to inform disease forecasting models. In order to accurately predict the future, mathematical models must account for the complex and interacting dynamics of the changing environment, the vectors (e.g., mosquitoes), and the host (e.g., the humans) based on past and current data. Specifically, real-time data on mosquito prevalence and diversity at various spatial resolutions is limited and often non-existent. Without these data streams, modelers use proxy information such as vegetation and hydrology to infer mosquito prevalence, however, these limitations increase the uncertainty in the predictions. Similarly, clinical surveillance data is often biased, due to limited and costly access, and delayed, due to the bureaucratic reporting process, taking weeks or even months to be published. In order to advance our ability to forecast diseases, we need billions of sensors around the world collecting and uploading real-time information to inform forecasting models.

The second major challenge to accurately forecasting infectious diseases is human behavior. Unlike weather, which is governed by the laws of physics, humans can be unpredictable and, as a host, they play a key role in the spread of infectious diseases. Human behavior, such as vaccination, can reduce or increase the probability of infection. Thus, understanding what people do during an epidemic is crucial for modeling disease dynamics. However, there are no databases monitoring human behavior and although Internet data streams, such as social media, have been used to capture population sentiment, there are biases and uncertainty in using this information. In addition, because of the role that humans are playing in changing the climate, it will be crucial to understand how their behavior will change model predictions. Finally, infectious disease forecasting needs significant investment in building the infrastructure necessary to collect data about the environment, the vectors, and the hosts at all spatial and temporal resolutions. These investments can also contribute to building a modeling community around the globe to support public health officials and ultimately reduce disease burden through forecasts with quantified uncertainty.

## 8. Concluding Remarks: What is Needed Now and in the Future for Forecasting the Impacts of Climate Change on Infectious Diseases

Rapid and large-scale environmental change is occurring globally, which is already leading to dramatic changes in animal, insect, and plant populations. While some wildlife species are able to adapt and others are not, infectious pathogens and their vectors will respond and take advantage of favorable situations. Additionally, as human populations move and expand, agricultural animals will continue to replace wildlife, and new hosts and vectors will move with these migrations and expansions. Coupling cutting-edge and validated climate models with scalable epidemiological models and ground-truth, real-time data will allow the models to be validated with statistical and sensitivity analyses in order to test hypotheses and predictions of future infectious disease conditions.

A focus on the potential impacts of climate change on natural, animal, and human systems will allow for better predictions and mitigations for how these impacts will influence zoonotic infectious diseases in animals and humans. While modeling has its limitations, especially when coupling large-scale systems models, the alternative includes guesses or waiting until infectious pathogens cross into new regions. Mitigations may include animal vaccination strategies that will take time to develop, including the time it takes to stockpile vaccines in areas where they are needed most. Having more knowledge of future vaccine requirements and the impact of animal trade and movement will ensure the best use of limited resources to reduce the threat of infectious diseases globally.

## Figures and Tables

**Table 1:** Primary zoonotic arboviruses and other pathogens vectored by Dipterans. The taxon groups listed for viruses are at the family level. Taxon groups for vectors are at the genus level.

Pathogen	Taxon Group	Vectors	Animal Hosts	Range
Banna virus	Reoviridae	Mosquitoes (*Culex*, *Anopheles*, *Aedes*) [12]	Pigs, cattle, humans [12]	Southeast Asia, Indonesia [12]
Chikungunya virus	Togaviridae [13]	Mosquitoes (*Aedes*) [13]	Humans [13]	Central America, North America, South America, Oceania, central/southern Africa, southern/eastern Asia, western/central Europe [14]
Dengue virus	Flaviviridae	Mosquitoes (primarily *Aedes aegypti*) [15]	Humans [15] In vitro successful infection of amphibians, mammals, & reptiles [16]	North America, Central America, South America, Oceania, Europe, southern Asia, and Africa [17]
Dirofilaria spp. (Dirofilariasis)	Nematode	Mosquitoes (*Culex*, *Aedes*, *Anopheles*, *Mansonia*) [18]	Dogs, cats, humans [19]	Southern/central/eastern Europe, Middle East, eastern/southeastern/central Asia, southeastern North America [19]
Eastern Equine Encephalitis virus	Flaviviridae	Mosquitoes (*Culiseta, Culex*) [20]	Birds, humans, horses [20]	United States [21]
Indiana vesiculovirus	Rhabdoviridae	Sand flies (*Phlebotomus*, *Lutzomyia*), mosquitoes (*Aedes*), black flies (family *Simuliidae*) [22]	Equids, bovids [22]	North America, South America [22]
Japanese Encephalitis virus	Flaviviridae	Mosquitoes (*Culex*) [17]	Pigs, birds, horses, humans [17]	Western Europe, Russia, southern Asia, Oceania [17]
Plasmodium spp. (Malaria)	Protozoan	Mosquitoes (*Anopheles*) [23]	Humans, birds (carriers) [23]	United States, central/southern Africa, southeast Asia, southern Europe [23,24]
Mayaro virus	Togaviridae	Mosquitoes (*Haemagogus*) [25]	Humans [25]	Northern South America *tourists infected [25]
Murray Valley Encephalitis virus	Flaviviridae	Mosquitoes (*Culex*) [26]	Humans [26]	Australia, Northern Territories [26]
O’nyong’nyong virus	Togaviridae	Mosquitoes (*Anopheles*) [27]	Humans [27]	Western/central Africa [27]
Oropouche virus	Peribunyaviridae	Mosquitoes (*Coquillettidia*) [28]	Humans [29]	Central/northern South America, southeastern Central America [28]
Rift Valley Fever virus	Phenuviridae	Mosquitoes (*Culex*, *Aedes*, *Anopheles*, *Eretmapodites*, *Mansonia*, *Culicoides*, *Coquillettidia*) [30]	Ruminants (reservoir), humans [30], Bats [31]	Africa, southern Middle East [30]
Saint Louis Encephalitis virus	Flaviviridae	Mosquitoes (*Culex*) [32]	Birds [32], humans [33]	South America [33], North America [33]
Spondweni virus	Flaviviridae	Mosquitoes (*Culex*, *Aedes*) [34]	Humans [34]	Caribbean, southern Africa [34]
*Trypanosoma brucei* (Sleeping Sickness)	Protozoan	Tsetse fly (*Glossina*) [35]	Wild ungulates, ruminants, equids, dogs, humans [35]	Africa, Central America, South America [35]
Usutu virus	Flaviviridae	Mosquitoes (*Culex*) [36]	Birds, horses, humans [36], bats [37]	Central/southern Africa, central Europe [36]
Venezuelan Equine Encephalitis virus	Togaviridae	Mosquitoes (*Culex*) [38]	Horses, humans [38]	Southern North America, central America, northern/central South America [38]
Western Equine Encephalitis virus	Togaviridae	Mosquitoes (*Culex*) [39]	Birds, humans [39]	Western North America, South America, Cuba [39]
West Nile virus	Flaviviridae	Mosquitoes (*Culex*, *Aedes*) [40]	Birds (reservoir), equids, humans [40]	Africa, the Middle East, Oceania, North America, Central America, northwestern and southern/central South America, and Europe [40]
Yellow Fever virus	Flaviviridae	Mosquitoes (*Aedes aegypti*, *Haemagogus*) [41]	Humans [41]	Central America, South America, southern North America, Oceania, Europe, southern Asia, and Africa [17]
Zika virus	Flaviviridae	Mosquitoes (*Aedes*) [42]	Primates, humans [42]	central-western and southern Africa, Oceania, South America, North America, Central America, southern Asia, Europe [17]

**Table 2:** Primary animal (non-zoonotic) pathogens vectored by Dipterans. The taxon groups listed for viruses are families. Taxon groups for vectors are at the genus level.

Pathogen	Taxon Group	Vectors	Animal Hosts	Range
African Horse Sickness virus	Reoviridae	Midges (*Culicoides*) [43]	Equids [43]	Central/southern Africa [43]
Bluetongue Disease virus	Reoviridae	Midges (*Culicoides*) [44]	Ruminants [44]	North America, Central America, South America, Africa, southern Asia, northern Australia, Estonia and Russia, southern and central Europe [45]
Epizootic Haemorrhagic Disease virus	Reoviridae	Midges (*Culicoides*) [46]	Ruminants [46]	North America, Australia, Africa, Asia, and the Mediterranean [46]
Lumpy Skin Disease virus	Poxviridae	Mosquitoes (*Aedes Anopheles*), flies (*Musca*, *Stomoxys*, *Glossina*), and midges (*Culicoides*) [47]	Cattle [47]	Africa, the Middle East, South-Eastern Europe, Russia [47]
Schmallenberg virus	Peribunyaviridae	Midges (*Culicoides*) [48]	Ruminants [49]	Europe [49]
*Trypanosoma evansi* (Surra)	Protozoan	Hematophagous flies (*Tabanus*, *Musca*) [50]	Vampire bats (reservoir), ungulates, ruminants, dogs, cats [50]	Asia, northern Africa, Central America, South America, the Middle East [50]
